# Votive Reliefs of Lower Extremities Disorders: A Brief Review

**DOI:** 10.7759/cureus.7020

**Published:** 2020-02-17

**Authors:** Efi Theodoraki, George K Paraskevas, Konstantinos Natsis, Niki Papavramidou

**Affiliations:** 1 Archaeology, Aristotle University of Thessaloniki, Thessaloniki, GRC; 2 Orthopaedics, Aristotle University of Thessaloniki, Thessaloniki, GRC; 3 Anatomy and Surgical Anatomy, Aristotle University of Thessaloniki, Thessaloniki, GRC; 4 History of Medicine, Aristotle University of Thessaloniki, Thessaloniki, GRC

**Keywords:** votive reliefs, lower extremity, graeco-roman period

## Abstract

The purpose of this paper is to present a series of votive reliefs from Graeco-Roman antiquity related to disorders of the lower extremities, in order to comprehend the importance of anatomical offerings as gifts of gratitude to healing gods such as Asclepius. The selected offerings cover disorders such as varicose vein disease and deformities of the lower limbs and provide significant information on medical treatments in ancient Greece, as well as the importance of religious practices in the healing process.

## Introduction and background

During the fifth century B.C. in ancient Greece, medical practice and research appeared to be highly developed. Professional physicians, such as Hippocrates or Galen, searched for the causes of the diseases they treated and promoted the systematic study of clinical medicine by directly examining living human beings or even attempting to perform various types of surgery [1-2]. However, despite this rational approach to treatment, formal medical practice was combined with a wide range of alternative options, such as prayers, charms, herbs, and diets, thus constituting a type of medical pluralism [3]. Out of all these folk medical treatments, the most popular was temple medicine with the sanctuaries of Asclepius as the most prominent option [4]. In these shrines, dream healing, known as incubation, along with prayer, were combined with classic Hippocratic treatment methods, including bloodletting, baths, diet, exercise, drugs, poultices, and emetics [5].

As a token of gratitude toward the healing god, patients offered a wide range of votive reliefs, depending on the body part that received the divine medical treatment. During excavations, archaeologists have recovered anatomical votive replicas of many body parts, both at Asclepian healing temples and other shrines related to healing gods. This paper focuses on the votive reliefs of patients with lower extremities disorders by presenting a number of archeological findings associated with such ailments.

## Review

For the analysis of the main subject, three anatomical reliefs were selected. All three can be dated back to Graeco-Roman antiquity, ranging from the fourth century B.C. to the early years of the first century A.D. Each of the sculptures presents lower limb areas with various forms of disorders in rather impressive detail. These three votive reliefs were examined in terms of imagery, text, the purpose of use, as well as disease, which may be depicted.

**Figure 1:**
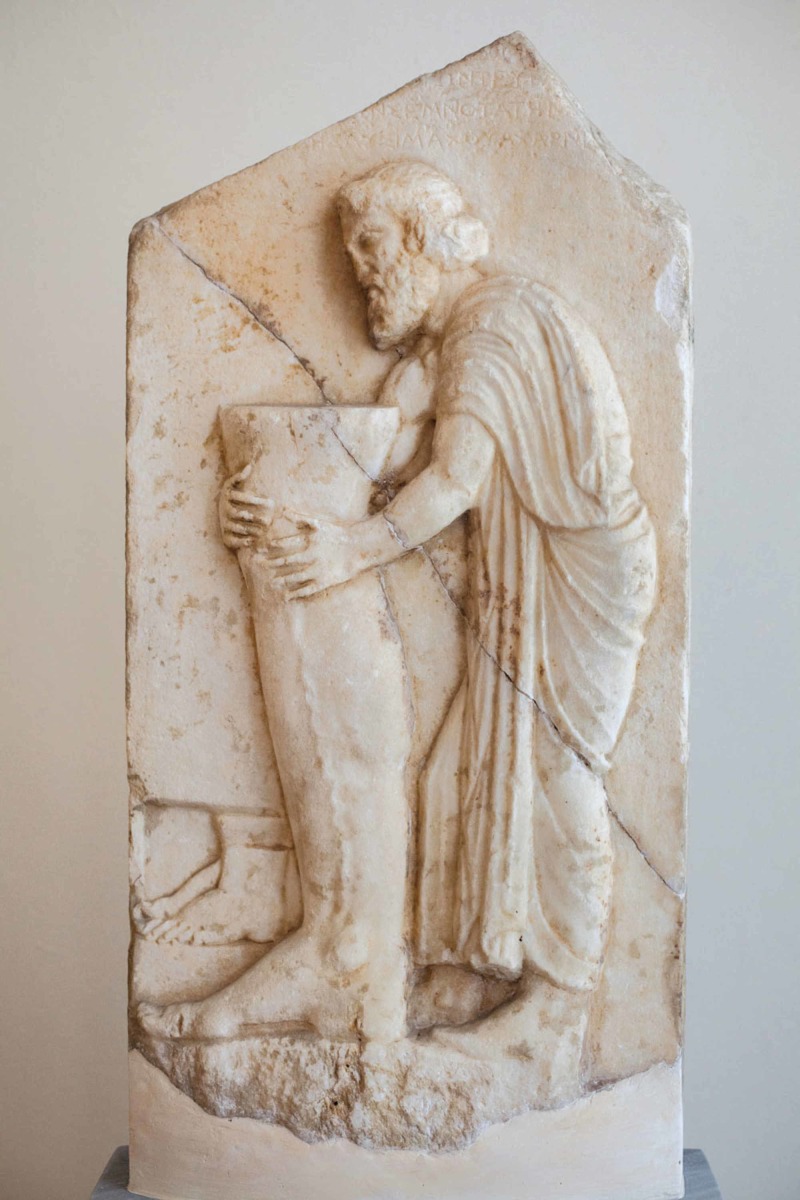
Votive relief found near Enneakrouno, Athens Part of the sanctuary of the hero-physician Amynos. The dedicator is holding the effigy of a leg. From fourth-century B.C., located in the National Archaeological Museum. Exhibit number 3256. © Hellenic Ministry of Culture and Sports

The votive relief of Figure 1 was found during excavations near the area of Enneakrouno, Athens. It is dated back to the end of the fourth century B.C., and it is part of the sanctuary dedicated to the hero-physician named Amynos. In the image, the dedicator appears to be offering the effigy of a leg with a swollen vein, a detail that could imply the disease from which he was suffering.

**Figure 2:**
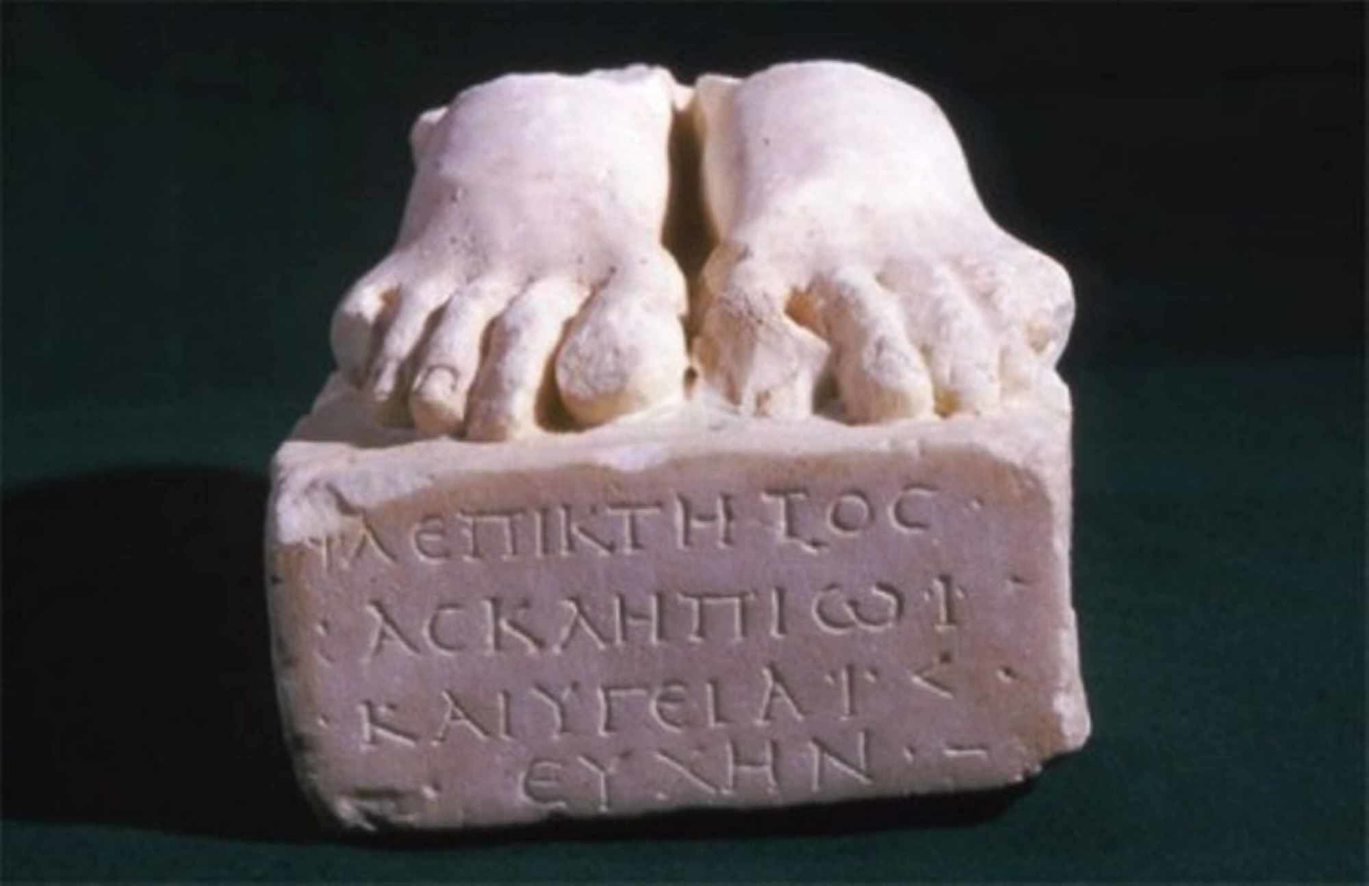
Votive sculpture dedicated to Asclepius and Hygeia. Part of the Asclepeion of Acropolis It depicts two feet standing on a stone basis. From the first century A.D. Located in the permanent collection of the Epigraphic Museum of Acropolis. Exhibit number 8419. © Museum of Acropolis

Another votive relief representing lower limb disorders was found at the Asclepieion of Acropolis, one of the most iconic sanctuaries devoted to the healing god during antiquity. This particular marble sculpture presented in Figure 2 was located in Athens, in the sanctuary of Asclepius on the south slope of the Acropolis and west of the Dionysus theater [5]. It depicts two feet placed on a stone base inscribed with ancient Greek writing. The inscription, which is written across four lines, reads that Flavius Epiktetus devotes the work to Asclepius and Hygeia. This votive relief dates back to the early Roman period (approximately first-century A.D.) and was probably offered by a patient to the two healing gods as a gift for the treatment of an unspecified foot disorder.

It is important to stress that, apart from Asclepius, the patient also turns to Hygeia for relief from his ailment. According to Greek mythology, Hygeia was the daughter of Asclepius and was considered to be a continuation of her father by participating in all the healing rituals and procedures that took place in his shrines. For this reason, she was considered a deity and patients would call upon her for the improvement of their health [6].

**Figure 3:**
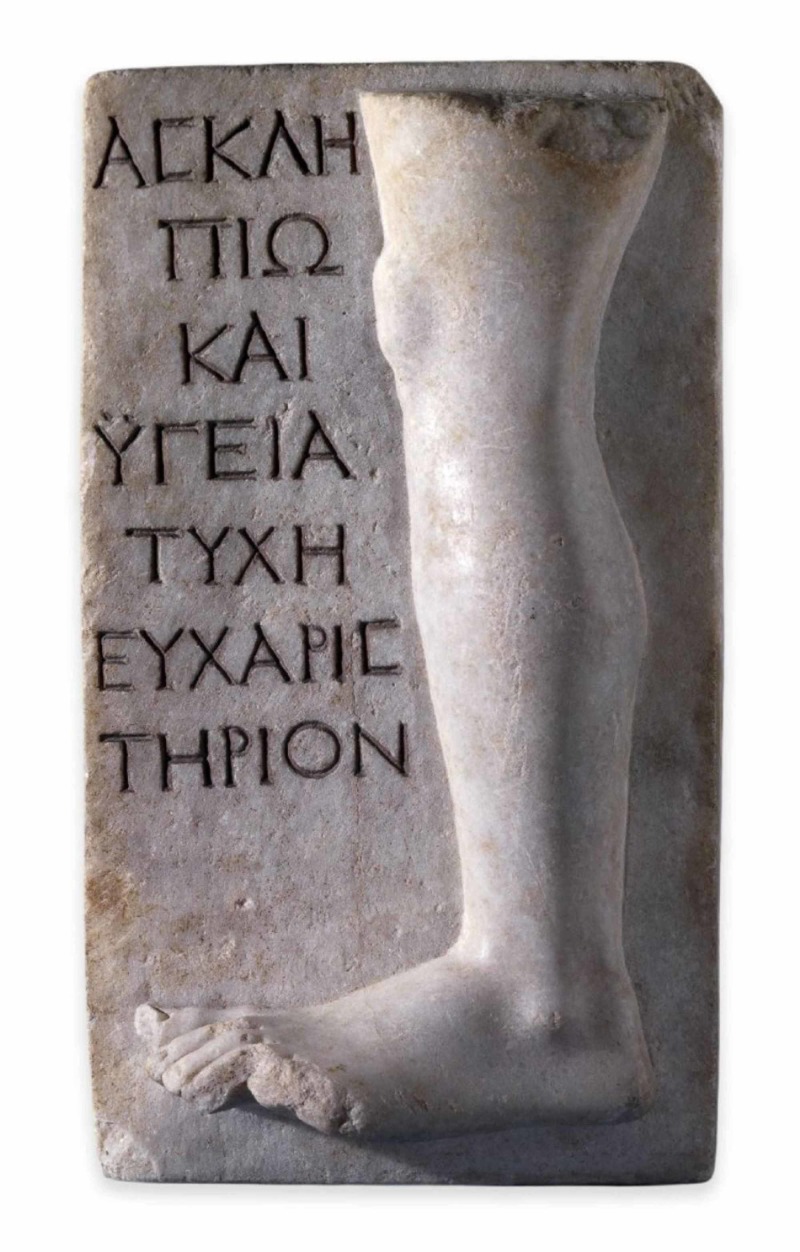
Votive relief dedicated to the healing gods. Part of the Asklepion of Milos It presents part of a left leg with severed toes. From 100-200 B.C. Located in the British Museum. ©Trustees of the British Museum.

The third and final votive relief related to lower limb disorders is that of Figure 3, which was found during an 1828 excavation in the shrine of Asclepius on the Cycladic island of Melos and is currently located in the exhibition of the British Museum. Dating back to a period between 100-200 B.C., the marble relief represents part of a leg with a number of severed toes, accompanied by an inscription. Just like the previous votive sculpture, it is devoted to both Asclepius and Hygeia, this time from a patient named Tyche as a thank-offering for the cure of an affliction of the left leg [7]. Based on the details of the anatomic relief, it can be assumed that the treatment applied in this case was a type of surgery for the necessary removal of the toes, due to either chronic obstructive arteriopathy, induced by diabetes or hypertension or a malformation such as syndactyly or traumatic injury or the possession of six toes. The late case seems to be more likely since the two outer toes, thus the fifth and sixth ones, are amputated. The first to fourth toes seem to be intact. We excluded the case of partial destruction of the sculpture at the site of the forefoot since the relative anatomic area seems to be well-designed and not destroyed.

As regards the enlarged big toes of the two feet, the edema seen may be due to gout arthritis or foot gout. As it is well-known, gout is a form of inflammatory arthritis induced by persistently high levels of uric acid in the blood and presented clinically by attacks of a red, tender, hot, and swollen joint, commonly that at the base of the big toe [8]. It is one of the oldest known diseases and has been first identified by the Egyptians as early as in 2460 B.C. Later in the fifth century B.C., Hippocrates referred to that disease as “the unwalkable disease” mentioning that clinical entity is “the arthritis of the rich.” Six centuries later, Galen was the first physician to describe tophi and recognize a hereditary trait [9]. We consider that the big toes of both feet of the marble sculpture found at the Asclepieion of Acropolis and, especially, the big toe of the right foot is remarkably enlarged and edematous at the area of its base, a condition resembling likely to the pathologic condition known as gout or podagra.

Based on the details of the anatomic relief seen in Figure 3, it can be assumed that the treatment applied in this case was a type of surgery for the necessary removal of the toes, likely due to the possession of six toes, and especially the fifth and sixth one, as is demonstrated on the sculpture of Figure 3. We consider from the morphology of the forefoot that the abovementioned toes are amputated and not destroyed either from traumatic injury or from sculpture damage.

Historical evidence of amputation has been obtained in Neolithic Neanderthal man as well on Peruvian votive figurines, with most of these amputations being minor in extent. The amputation of limbs was widely known by Hippocrates (460-377 B.C.) as the therapeutic method, especially when a gangrenous part of the limb was needed to remove. Later on, Celsus (25 B.C. to 50 A.D.), suggested the application of a circular amputation technique [10].

All the aforementioned votive offerings provide important information regarding the treatment of afflictions of the lower extremities in the Graeco-Roman world. The commonality and severity of certain types of disorders can be identified both from anatomical reliefs and other texts from the same period. For instance, the treatment of varicose veins depicted in Figure 1 can be associated with the work of ancient medical practitioners in the field of venous hypertension [11]. The marble table from ancient Athens that is demonstrated in Figure 1 can be one of the first depictions of varicose vein disease and its purpose was most likely to act as a gift from the grateful patient toward the deity. The varicose veins are mentioned very early in Ebers Papyrus in which any relative surgical procedure is not allowed with the notice “thou shall not touch something like this” [2]. Hippocrates and other significant Graeco-Roman physicians, such as Celsus and Galen, adopted a methodology for the treatment of varicose veins, which does not directly contradict contemporaneous knowledge on the issue [12]. More specifically, Hippocrates recognized a link between the ulcers appearing on the leg and recommended the use of double-bandage compresses combined with the application of various herbs [13]. In the case of varicose veins, Hippocrates opposed to surgery and limited the application only to minuscule incisions supplemented by compresses [7]. In specific, in the latter cases, Hippocrates performed cauterization of the varicose veins with a hot iron [14]. Later on, Aurelius Cornelius Celsus (53 B.C. to 7 A.D.) executed small incisions and removed parts of varicose veins after cauterization, whereas Claudius Galenus (130-200 A.D.) removed parts of the vein between ligatures using hooks [15].

The varicose vein disease treatments developed by the physicians of the fifth century B.C. and the Hellenistic period were adopted later on by the Byzantines and western medical practitioners. From the early days of the empire, Byzantine physicians focused strongly on the improvement of varicosis surgery [16]. It is characteristic that compression, marking, ligation, and stripping of the great saphenous vein were practiced by a Byzantine physician named Paulus Aegineta (607-690 A.D.) [17].

## Conclusions

The votive reliefs of lower extremity disorders presented in this paper are only a few of the many found across the ancient Mediterranean world. By studying these anatomical reliefs, significant evidence can be extracted on two main aspects of ancient Greek life. On the one hand, the evolution of professional medical development can be comprehended thanks to particular details depicted on the limbs presented on the reliefs. On the other hand, these gifts of gratitude show the important social role played by religious medical sanctuaries, mainly those of Asclepius, which patients saw as a source of hope to turn to during their search for a cure to their disease.
